# Bipolar hip arthroplasty as salvage treatment for loosening of the acetabular cup with significant bone defects

**DOI:** 10.3205/iprs000092

**Published:** 2016-04-04

**Authors:** Mohamed Ghanem, Almuth Glase, Dirk Zajonz, Andreas Roth, Christoph-E. Heyde, Christoph Josten, Georg von Salis-Soglio

**Affiliations:** 1 Department of Orthopaedic Surgery, Traumatology and Plastic Surgery, University Hospital Leipzig, Leipzig, Germany

**Keywords:** bipolar hip arthroplasty, revision surgery, salvage procedure

## Abstract

**Introduction:** Revision arthroplasty of the hip is becoming increasingly important in recent years. Early primary arthroplasty and longer life expectancy of the patients increases the number of revision surgery. Revision surgery of hip arthroplasty is major surgery for the patients, especially the elderly, with significant risks concerning the general condition of the patient. The aim of this work is to evaluate the outcome of bipolar hip arthroplasty as a salvage procedure for treatment of loosening of the acetabular cup with significant acetabular bone defects after total hip replacement (THR) in multi-morbid patients.

**Patients and methods:** During the period from January 1^st^ 2007 to December 31^st^ 2011 19 revision hip surgeries were performed in 19 patients, in which the loosened acetabular cup was replaced by a bipolar head. The examined patient group consisted exclusively of female patients with an average of 75 years. The predominant diagnosis was “aseptic loosening” (84.2%). All patients in our study were multi-morbid. We decided to resort to bipolar hip arthroplasty due to the compromised general condition of patients and the major acetabular bone defects, which were confirmed intraoperatively. The postoperative follow-up ranged from 0.5 to 67 months (average 19.1 months).

**Results:** Evaluation of the modified Harris Hip Score showed an overall improvement of the function of the hip joint after surgery of approximately 45%.

Surgery was less time consuming and thus adequate for patients with significantly poor general health condition. We noticed different complications in a significant amount of patients (68.4%). The most common complication encountered was the proximal migration of the bipolar head.

The rate of revision following the use of bipolar hip arthroplasty in revision surgery of the hip in our patients was high (21%). Despite the high number of complications reported in our study, we have noticed significant improvement of hip joint function as well as subjective pain relief in the majority of patients. We clearly achieved clinically satisfactory results in 14 patients.

**Conclusion:** Bipolar hip arthroplasty is by no means to be regarded as standard procedure in revision surgery of THR. It provides an option or salvage procedure for patients with poor general condition in whom the quickest possible surgical intervention preserving mobility is required. This is particularly true for multi-morbid patients in whom sufficient acetabular fixation is not possible.

## Zusammenfassung

**Einleitung:** Die Anzahl der Revisionsendoprothetik am Hüftgelenk steigt durch Zunahme der frühen Erstimplantationen und der längeren Lebenserwartung der Patienten. Die Revisionsoperation ist mit einem hohen operativen Aufwand verbunden und bedeutet für den Patienten ein nicht unerhebliches Risiko.

Ziel dieser Arbeit ist die Evaluierung der Duokopfendoprothese als Rückzugsmöglichkeit bei Pfannenlockerung mit acetabulären Knochendefekten bei multimorbiden Patienten.

**Patienten und Methode:** Im Zeitraum vom 1. Januar 2007 bis 31. Dezember 2011 wurden 19 Revisionsoperationen bei 19 Patienten durchgeführt, bei denen eine Pfanne gegen eine Duokopfendoprothese ausgetauscht wurde. Die untersuchte Patientengruppe bestand ausschließlich aus weiblichen Patienten, das Alter der Patientinnen lag zum Zeitpunkt der Wechseloperation auf eine Duokopfendoprothese im Mittel bei 75 Jahren. Die häufigste Diagnose war aseptische Lockerung (84,2%). Alle Patientinnen unserer Studie waren multimorbide. Die Entscheidung zu der Duokopfendoprothese als Sekundärendoprothese wurde aufgrund der Multimorbidität bei intraoperativ desolatem knöchernen Befund der Hüftpfanne getroffen. Die postoperative Nachbeobachtungszeit betrug zwischen 0,5 und 67 Monaten (Durchschnitt 19,1 Monate).

**Ergebnisse:** Das Gesamtergebnis der operativen Versorgung an Hand des modifizierten Harris Hip Score ergab eine Verbesserung der Funktionalität von ca. 45%.

Die Operationen waren weniger zeitaufwendig und damit für Patienten mit signifikant schlechtem Gesundheitszustand angemessen. Bei vielen Patienten (68,4%) sind verschiedene Komplikationen aufgetreten. Die häufigste Komplikation war die proximale Migration des Duokopfes.

Trotz der hohen Anzahl von Komplikationen in unserer Studie konnten wir eine signifikante Verbesserung der Hüftgelenksfunktion sowie subjektive Schmerzlinderung bei der Mehrzahl der Patienten verzeichnen. Bei 14 Patienten wurden klinisch zufriedenstellende Ergebnisse erreicht. Dennoch war die Revisionsrate in unserem Patientengut hoch (21%).

**Fazit:** Die Duokopfendoprothese ist keineswegs als Standardverfahren bei der operativen Behandlung von Pfannenlockerungen zu betrachten. Sie stellt eine Option bzw. Rückzugsmöglichkeit bei multimorbiden Patienten dar, bei denen die schnellstmögliche, aber mobilitätserhaltende operative Intervention erforderlich ist, dar. Insbesondere gilt dies für multimorbide Patienten, bei denen eine erneute Pfannenfixation auf Grund der erheblichen knöchernen Defekte nicht erfolgversprechend mit vertretbarem Aufwand möglich ist.

## Introduction

Revision arthroplasty of the hip is becoming increasingly important in recent years. With an average durability of implants of more than ten years [11], [12] early primary arthroplasty and longer life expectancy of the patient increases the number of revision surgery [2], [13], [14], [23].

Almost 210,000 total hip replacements (THR) are carried out annually in the Federal Republic of Germany, the number of revision surgery on artificial hip joint being approximately 25,800 [1].

The main reason for failure of a primary THR is aseptic loosening, the acetabular component being affected twice as often as the stem [5], [24]. Other causes may be septic loosening, periprosthetic fractures and dislocations [10], [16].

Revision surgery of hip arthroplasty is major surgery for the patient, especially the elderly, with significant risks concerning the general condition of the patient. Thus operation time should be kept as short as possible and blood loss can be kept low in order to reduce the risk of peri- and postoperative morbidity and mortality [19]. Girdlestone situation is considered as a last resort in certain situations such as persistent infection or patients with significantly compromised general condition [17], [21]. Patients with Girdlestone situation usually don’t complain of pain, however the function of the hip joint is compromised [17], [21].

The outcome of acetabular revision is critically dependent on the initial situation of the bony acetabulum. In case of large bony defects the stability of an acetabular implant becomes difficult.

Several implants are available for revision of the acetabular cup. In order to obtain an optimum result, stability of the implant must be ensured, which prevents migration, dislocation or a renewed loosening. In addition, the center of rotation should be re-constructed. Bone defects can be filled with bone grafts and/or supplied by the proper acetabular implant in order to ensure stable anchorage. Overall, the results of the various revision surgeries and implants of hip arthroplasty are difficult to compare because of the diverse classifications of bone-defects and the insufficient classification of the initial clinical findings in literature [7], [8].

In our clinic, bipolar head was used as a salvage procedure in revision hip arthroplasty for loosening of the acetabular cup after THR. This treatment strategy was performed in 19 patients between 2007 and 2011. The indication was provided when a stable fixation of the revision-acetabular cup was unlikely either due to major intraoperative risks concerning the general condition of the patient or large acetabular bone defects.

The present study evaluates the pre-, intra- and postoperative course of these patients. The aim of this work is to evaluate the outcome of bipolar hip arthroplasty as a salvage procedure for the treatment of loosening of the acetabular cup with significant acetabular bone defects after THR in multi-morbid patients.

## Patients and methods

We recorded all revision surgeries of the acetabular cup which were treated by bipolar hip during the period from January 1^st^ 2007 to December 31^st^ 2011 at the department of orthopedic surgery, University of Leipzig. Collecting patient data was carried out based on Electronic Health Records in SAP IS-H (Siemens AG Healthcare Sector, Erlangen, Germany) as well as archived patient records.

Clinical and radiological follow-up examination was carried out in February 2012 in the department of orthopedic surgery, University of Leipzig. 19 revision hip surgeries were performed in 19 patients, in whom the loosened acetabular cup was replaced by a bipolar head.

The examined patient group consisted exclusively of female patients in whom the common preoperative diagnosis was relevant loosening of the acetabular cup after THR. The age of the patients at the time of revision surgery ranged from 55–89 years with an average of 75 years. In 16 patients the main diagnosis (84.2%) was “aseptic loosening”. In two out of those 16 patients, recurrent dislocation was reported in addition.

In two patients (10.5%) the diagnosis was “septic loosening”.

In one case (5.3%) the indication was provided as a result of traumatic periprosthetic acetabular fracture. 

At this point it should be noted that 17 patients in our study (89.5%) were multi-morbid and were classified according to the American Society of Anesthesiologists as ASA 3. We decided to resort to bipolar hip arthroplasty due to the compromised general condition of the patients and the major acetabular bone defects, which were confirmed intraoperatively. The remaining 2 patients (10.5%) were classified as ASA 2, but had extensive acetabular bone defects which could have only been treated by a custom made endoprosthesis.

The postoperative follow-up ranged from 0.5 to 67 months (average 19.1 months). Statistical evaluation was carried out using spreadsheet software, Microsoft Excel (Microsoft Corporation, Redmond, USA).

The surgical approaches used were the anterolateral or lateral transgluteal hip approach. The loosened acetabular cup was removed. Radical synovectomy and debridement were performed. The acetabular bony structure was evaluated. Careful evaluation of the stability of the femoral stem was carried out. The bipolar head was implanted after prior intraoperative clinical and radiological evaluation, especially concerning size and stability.

On follow-up, we conducted clinical and radiological examination and recorded the modified Harris Hip Score [9].

## Results

The preoperative modified Harris Hip Score values ranged from 27 to 45 points with an average of 36.2 points. The evaluation of the modified Harris Hip Score after discharge from hospital revealed a range from 29–90 points with an average of 52.3 points. This resulted in an overall improvement of the functionality of approximately 45%.

The duration of the surgical intervention ranged from 73 up to 226 minutes (average: 148 minutes). 

**Figure 1:**
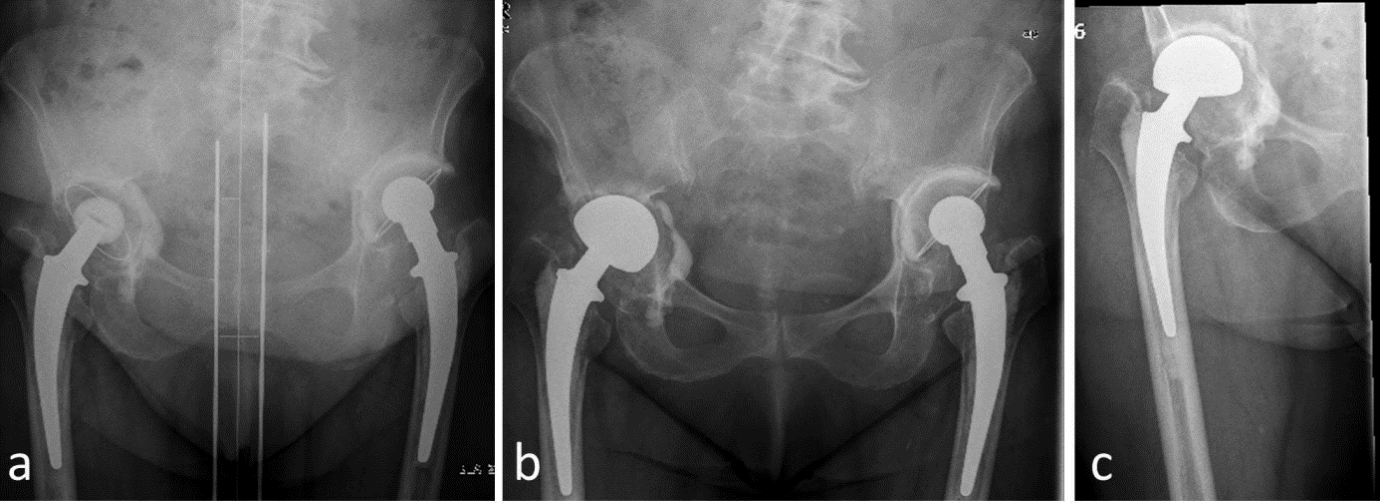
Figure1: 72-year-old patient, ASA 3, intraoperative significant acetabular bone defects. Uncomplicated postoperative course. Improvement of the Harris Hip Score from 39 preoperatively to 74 two years after revision surgery. a. aseptic loosening of the acetabular cup, b. immediate postoperative result, c. two years after surgery

In 6 patients (31.6%) no complications were recorded at all (Figure 1 (Fig. 1)). On the contrary, we noticed different complications in the remaining 12 patients (68.4%). These are to be listed in detail:

In 8 patients (42.1%), there was one single complication reported, in 5 patients (26.3%) we noticed several complications.

**Figure 2:**
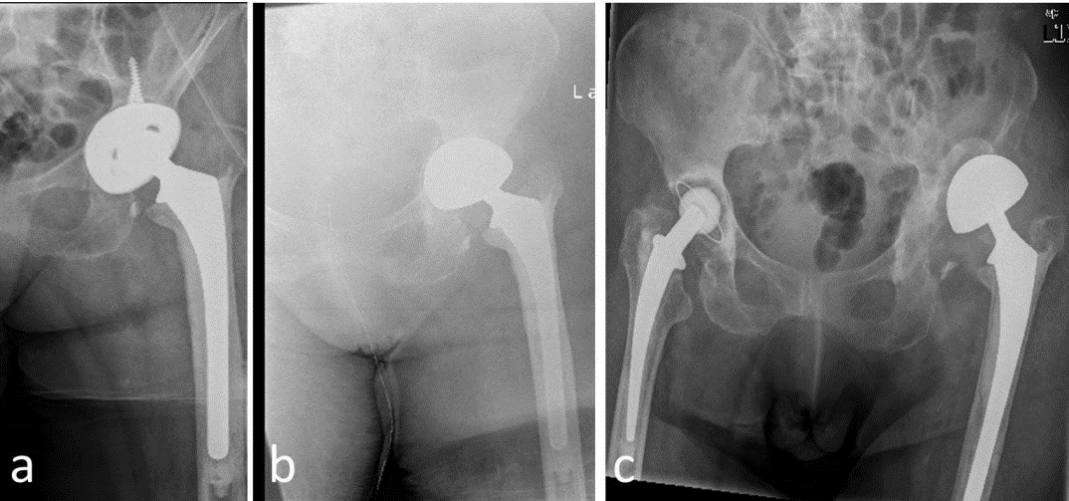
Proximal migration of the bipolar head. a. aseptic loosening of the acetabular cup, b. immediate postoperative result, c. one year after surgery showing proximal migration

The most common complication encountered was the proximal migration of the endoprosthesis (Figure 2 (Fig. 2)) that we have observed in 7 (36.8%) patients, followed by dislocation in 6 cases (31.6%).

In 4 patients (21%), cranial migration was the single complication encountered. In 3 patients no further surgical measures were necessary. In the 4^th^ patient we observed loosening of the stem 2 years later after revision surgery. Major revision surgery was contraindicated due to the deteriorated general condition. Therefore, explantation of the endoprosthesis (Girdlestone) was carried out.

Dislocation as the sole complication occurred in 3 (15.8%) patients. In all 3 cases we noticed recurrent dislocations that have been treated by closed reposition. One patient declined further surgical treatment due to the fact that she had been operated on several times before. In one patient Girdlestone has been carried out. In the third patient, a change to a larger head has been carried out.

4 patients (21%) experienced an infection. In 2 patients infection healed after surgical revisions and the artificial hip joint could be saved. In 2 patients, however, explantation was needed ending in a Girdlestone situation.

In one patient (5.3%) a partial paralysis of the peroneal nerve occurred on the contralateral side. This was treated conservatively and recovered significantly during the further course.

One patient (5.3%) suffered pelvic fracture during the further course and was treated conservatively.

In one patient (5.3%) we encountered intraoperative femoral shaft fracture. There was no sign of loosening of the shaft. Therefore, the fracture was sufficiently treated with cerclage.

An overview of the preoperative condition of our patients as well as the duration of the surgical procedure is illustrated in Table 1 (Tab. 1). The complications encountered are shown in Table 2 (Tab. 2).

## Discussion

Bipolar hip arthroplasty is distinguished from THR by a shorter operative time, less blood loss and reduced risk of postoperative dislocation [3], [6]. This endoprosthesis is primarily used for fractures and necrosis of the proximal femur, mainly in femoral neck fractures [4]. Few reports exist in literature on the use of bipolar hip arthroplasty in revision surgery of the hip. Scott published a study in 1985 on the use of bipolar arthroplasty in revision surgery of the hip associated with bony reconstruction [22]. Hemiarthroplasty was used here as interim endoprosthesis and should be removed after bony healing. A major number of these patients, however, was so pleased with the interim endoprosthesis that no further surgery was performed [22]. In a study performed in 1991 a combination of bony reconstruction and bipolar hip arthroplasty was applied in patients with major acetabular bony defects [15]. Here, it was noted that the majority of implants has shown proximal migration over time. The rate of revision surgery amounted to 13.3%. Better results, however, were reported by Roberson et al. [20]: from 25 patients who were treated with bipolar hip and additional cancellous allografts which were implanted in large acetabular defects, two patients showed cranial migration during the further course. In one case there was an acetabular bone defect with dislocation and in the further case a periprosthetic fracture was demonstrated.

In 2000, a study on the use of bipolar endoprostheses as a treatment for hip joint instability after THR was performed by Parvizi et al. [18]. In 81% of the cases further dislocations were not reported, in 26% further revision surgery proved to be necessary.

In hip surgery bipolar arthroplasty is almost exclusively employed in cases with femoral neck fractures. In patients with poor general condition and correspondingly increased risk of surgery the shortest time of surgical intervention and surgical induced trauma with the ability of a mobility preserving procedure is of fundamental importance. In loosening of the acetabular cup with significant bone defects of the acetabulum, revision surgery to implant a suitable revision acetabular cup is undoubtedly the surgical treatment of choice [7], [8], [18], [20], [23]. However, these procedures are time consuming and therefore generally associated with higher risk of intra- and postoperative complications. 

In cases with significant acetabular bone defects, conventional acetabular revision implants might still be insufficient. In order to provide adequate stability of the acetabular implant, the surgical procedure entails the prior preparation of a custom made implant, which comprises high costs, in addition to a significant prolongation of the duration of surgical procedure. On the contrary, the use of bipolar hip arthroplasty in revision surgery of the hip can be performed relatively quickly. In our study, the duration of surgery ranged from 73 up to 226 minutes (average: 148 minutes). In all patients, in whom the duration of the intervention was longer than 120 minutes, the implantation of a revision acetabular cup was first attempted and proved to be very complicated and time consuming and thus exposing the patients to much higher perioperative risks. In the comparable studies no precise information on the operating time was documented [15], [18].

Therefore, we conducted this study to verify, whether the indication for bipolar hip arthroplasty can be justified in special cases of revision surgery of THR.

The collected data show a high rate of complications. In 6 patients (31.6%) no complications were registered at any time. In contrast, the remaining 12 patients (68.4%) showed different complications, 5 of them (26.3%) werethen treated conservatively.

Despite the high number of complications reported in our study, we have noticed significant improvement of hip joint function as well as subjective pain relief in the majority of patients. We clearly achieved clinically satisfactory results in 14 patients. The Harris Hip Score improved by an average of 45%. After revision surgery we recorded a mean Harris Hip Score in our patients of 52.3 points. Parvizi recorded an improvement in the Harris Hip Score with a mean of 55 in his comparable study [18]. At this point, however, it should be noted that the average age of patients in our study (75 years) was much higher than in the study of Parvizi (61 years) [18]. 

The most important parameters of improvement for the patient are pain relief, sufficient mobility and self-reliance. 88.9% of our patients claimed to have suffered from severe pain preoperatively. After revision surgery using bipolar hip arthroplasty, only 33.3% of our patients still experienced severe pain. 22.2% of our patients reported complete relief of pain after surgery.

The rate of revision following the use of bipolar hip arthroplasty in revision surgery of the hip in our patients was high (21%). Previous studies achieved results with lower rate of revision ranging from 8% to 13% [15], [18]. The better results are possibly due to the additional bone plasty of the acetabular roof, which was performed in the majority of cases.

## Conclusions

Surgical treatment of patients with poor general conditions who present with loosening of the acetabular cup and extensive acetabular bone defects after THR remains a major challenge. Bipolar hip arthroplasty is by no means to be regarded as standard procedure in revision surgery of THR. It provides an option or salvage procedure for patients with poor general condition in whom the quickest possible surgical intervention is required. This is particularly true for multi- morbid patients in whom sufficient acetabular fixation is not possible. Because of the high complication rate and rather moderate improvement of functionality, the use of bipolar hip arthroplasty in revision hip surgery after THR can only be recommended as a salvage procedure.

## Limitation

The retrospective design, small sample size and the lack of preoperative classification of the acetabular defects are limitations of this study. Universal criteria that apply on the indication for implantation of bipolar hip in revision arthroplasty of the hip cannot be derived from this study. Further studies in larger populations with prospective design are recommended.

## Notes

### Authorship

The authors Mohamed Ghanem and Almuth Glase contributed equally to this work.

### Competing interests

The authors declare that they have no competing interests.

## Figures and Tables

**Table 1:**
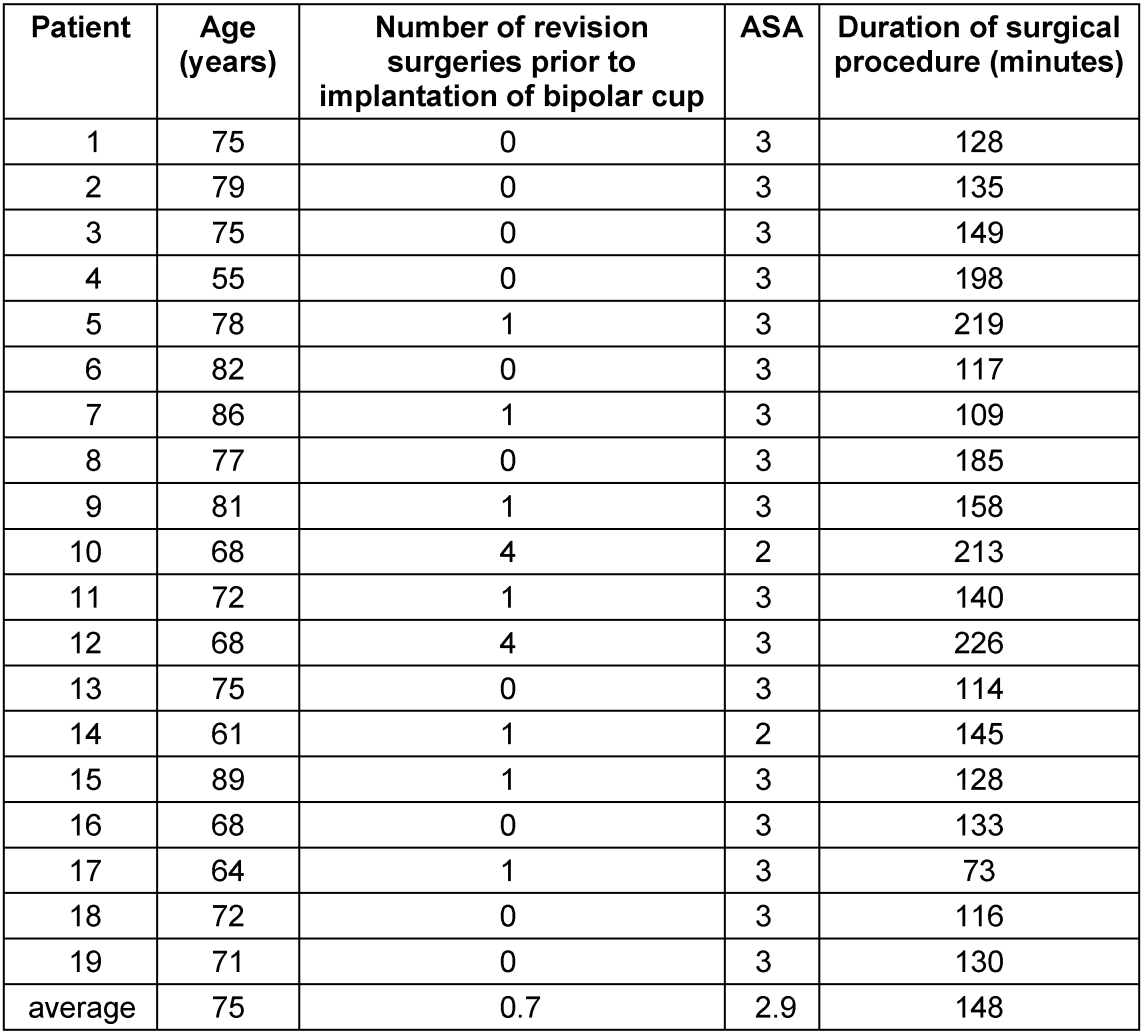
Preoperative condition of patients and duration of surgical intervention

**Table 2:**
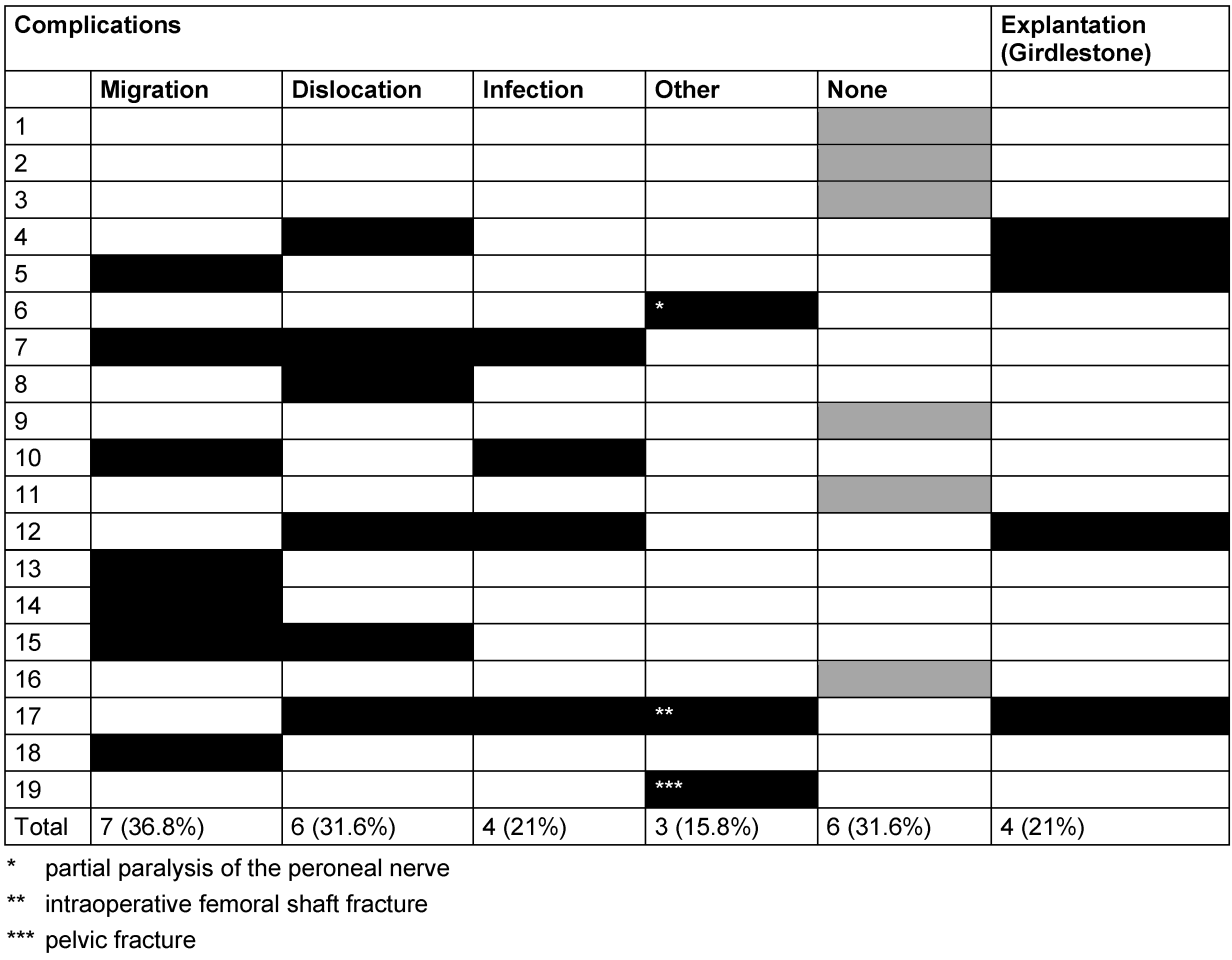
Complications
